# Application and Validation of Semiautomatic Quantification of Immunohistochemically Stained Sections for Low Cellular Tissue Such as Intervertebral Disc Using QuPath

**DOI:** 10.1002/jsp2.70054

**Published:** 2025-03-06

**Authors:** Andrea Nüesch, Maria Paola Ferri, Christine L. Le Maitre

**Affiliations:** ^1^ Division of Clinical Medicine, School of Medicine and Population Health University of Sheffield Sheffield UK; ^2^ Barcelona Supercomputing Center Barcelona Spain; ^3^ Universitat de Barcelona Barcelona Spain

**Keywords:** image analysis, immunohistochemistry, IVD, quantification, semi‐automated

## Abstract

**Background:**

Immunohistochemistry (IHC) is a widely used method for localizing and semi‐quantifying proteins in tissue samples. Traditional IHC analysis often relies on manually counting 200 cells within a designated area, a time‐intensive and subjective process that can compromise reproducibility and accuracy. Advances in digital scanning and bioimage analysis tools, such as the open‐source software QuPath, enable semi‐automated cell counting, reducing subjectivity and increasing efficiency.

**Aims:**

This project developed a QuPath‐based script and detailed guide for semi‐automatic cell counting, specifically for tissues with low cellularity, such as intervertebral discs and cartilage.

**Methods and Results:**

The methodology was validated by demonstrating no significant differences between the manual counting and the semi‐automatic quantification (*p* = 0.783, *p* = 0.386) while showing a strong correlation between methods for both collagen type II staining (*r* = 0.9602, *p* < 0.0001) and N‐cadherin staining (*r* = 0.9044, *p* = 0.0001). Furthermore, a strong correlation (intraclass correlation coefficient (ICC) single raters = 0.853) between 3 individual raters with varying academic ranks and experiences in IHC analysis was shown using the semi‐automatic quantification method.

**Discusssion:**

The approach ensures high reproducibility and accuracy, with reduced variability between raters and laboratories. This semi‐automated method is particularly suited for tissues with a high extracellular matrix to cell ratio and low cellularity. By minimizing subjectivity and evaluation time, it provides a robust alternative to manual counting, making it ideal for applications where reproducibility and standardization are critical. While the methodology was effective in low‐cellularity tissues, its application in other tissue types warrants further exploration.

**Conclusions:**

These findings underscore the potential of QuPath to streamline IHC analysis and enhance inter‐laboratory comparability.

## Graphical abstract

This study presents a semi‐automatic pipeline for quantitative analysis of immunohistochemically stained slides, particularly for low‐cellularity tissues such as intervertebral discs (IVDs) and cartilage. The semi‐automatic method developed using QuPath addresses key limitations of manual cell counting, such as human fatigue, subjective interpretation, and regional bias, improving both accuracy and reproducibility. High inter‐rater agreement and strong correlation with manual counting underscore the method's robustness and reliability.

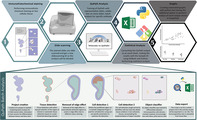

## 1 Introduction

Immunohistochemistry (IHC) is a standard technique used for the localization and semi‐quantification of protein detection [1]. IHC is extremely useful because it does not only preserve cell morphology and the tissue architecture, but is also highly sensitive, enabling the characterization of certain biomolecules at the protein level within the tissue [1]. The process involves applying a primary antibody that binds to a specific target protein. A secondary antibody, conjugated with either a fluorophore, enzyme, or biotin, then binds to the primary antibody, allowing for colorimetric analysis through substrate conversion. This enables the quantification of immunopositive and immunonegative cells [2].

The standard quantification of immunopositivity is to perform semiquantitative analysis by manually counting 200 cells within a region of interest [2]. However, manual scoring can be subjective and prevent reproducible and objective analysis essential for the quantification and correlation of proteins in biological tissue. Furthermore, by only counting 200 cells, regional variability may not be considered. With the advent of the ability to acquire high‐resolution digital scans of entire microscopic slides, combined with whole slide scanning and the use of new bioimage analysis tools such as QuPath, immunohistochemical analysis can be performed semi‐automatically using digital image analysis [3, 4, 5, 6].

QuPath is an open‐source software for bioimage analysis and is often used for digital pathology. Featuring a user‐friendly interface, embedded algorithms for tissue and cell detection, interactive machine learning capabilities, and the option for automated scripting, this offers a robust solution for analyzing whole slide images [5]. Even though QuPath has a built‐in algorithm for the detection of cells and their classification into immuno‐positive and negative cells, its accuracy is impacted by the cellularity of the tissue. In particular, tissues with low cellularity are impeded as tissue artifacts are wrongly detected as cells. Tissues considered to have a high extracellular matrix to cell ratio include tendons [7], ligaments [8], cartilage [9] and the components of the intervertebral disc [10].

Additionally, while QuPath facilitates image analysis, it lacks tools for streamlined post‐processing of large datasets. Users typically export data manually to platforms like Excel or R for further statistical analysis, a process that is labor‐intensive and prone to error. Addressing these limitations is critical for ensuring reproducibility and standardization in IHC analysis. To address this, within this program of work, we developed the ProcessScanningData class [11], a novel tool designed to automate the post‐processing of data from various whole‐slide image formats, including MRXS, NDPI, CID, and BIZ. This class integrates scanned image data with inventory metadata, calculates immunopositivity statistics, and produces detailed output files. Additionally, it generates visual outputs such as scatter plots and heatmaps, enabling efficient exploration of relationships between markers [12]. By automating these processes, the tool significantly reduces the need for manual intervention, improving accuracy and efficiency, especially in the analysis of tissues with low cellularity [13].

The project aimed to create a QuPath‐compatible script and an accompanying step‐by‐step guide tailored for semi‐automatic cell counting and classification of hematoxylin‐DAB (H‐DAB)‐stained slides, to specifically enable accurate determination of cellular immunopositivity. The methodology was designed to ensure reproducible immunopositivity rates with high accuracy, minimize inter‐rater and inter‐laboratory variability, and significantly reduce the time required for sample evaluation. The approach is particularly suited for the analysis of low‐cellularity tissues, such as intervertebral discs, cartilage, and bone, where traditional methods fall short.

## 2 Methods

### 2.1 Study Design

**FIGURE 1:**
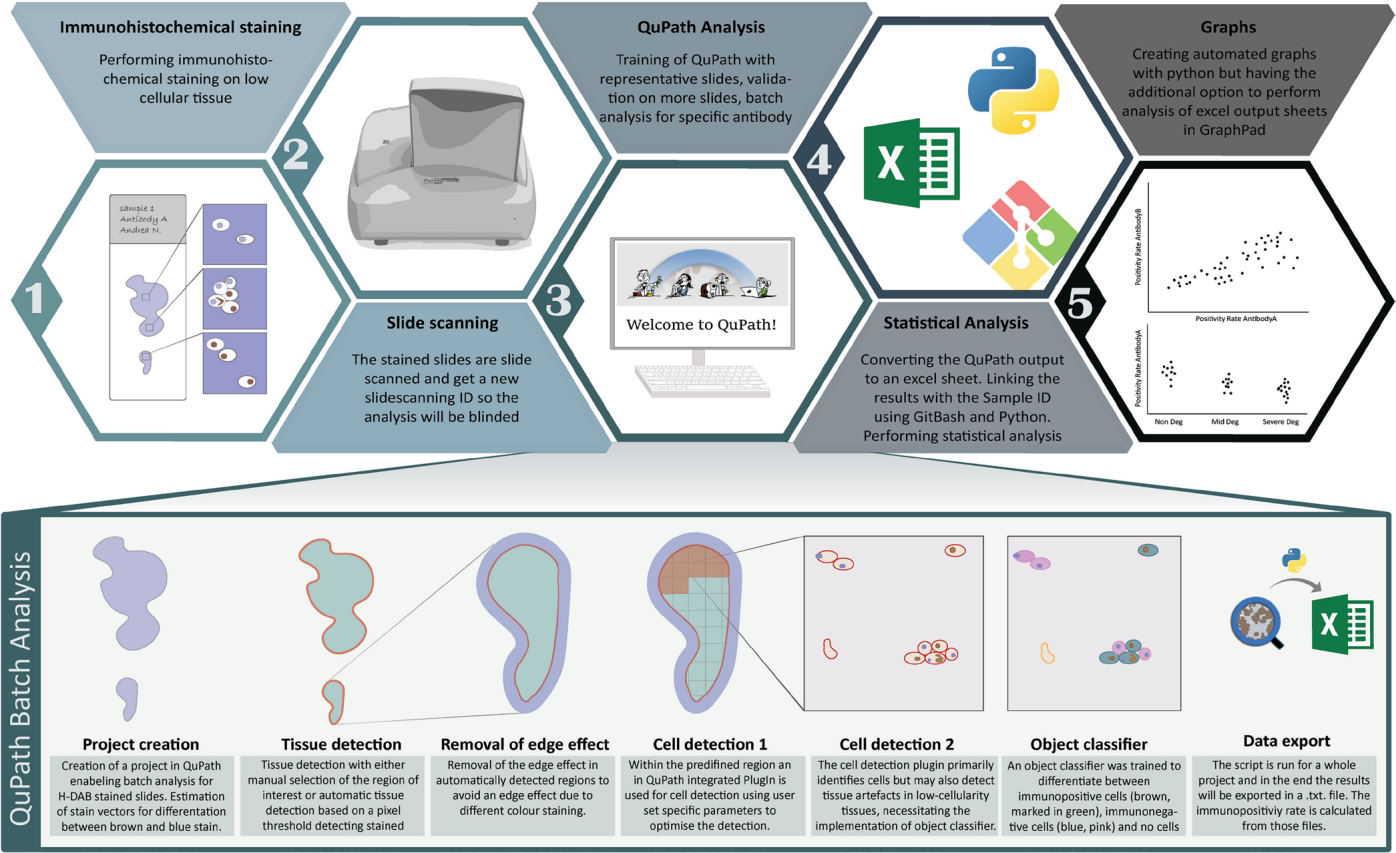
Analysis workflow. After immunohistochemical staining, the slides are scanned and analyzed using QuPath before proceeding with statistical analysis and graph creation. Prior to running the QuPath batch analysis, the system undergoes training, during which the Cell Detection parameters are defined, an object classifier is trained, and regions of interest are selected (Figure 2). Batch analysis is then performed within a QuPath project based on the pre‐defined parameters established during the initial training process. During this analysis, cells within the regions of interest are detected according to the pre‐set parameters and classified as either “positive cell,” “negative cell,” or “no cell.” The batch process analyzes all slides within the project and generates a folder containing text files with the results. In the subsequent step, a Python script consolidates these text files into a single Excel spreadsheet, which provides the positivity rate for each selected region of every slide. This consolidated data is then used for statistical analysis and visual representation.

**FIGURE 2:**
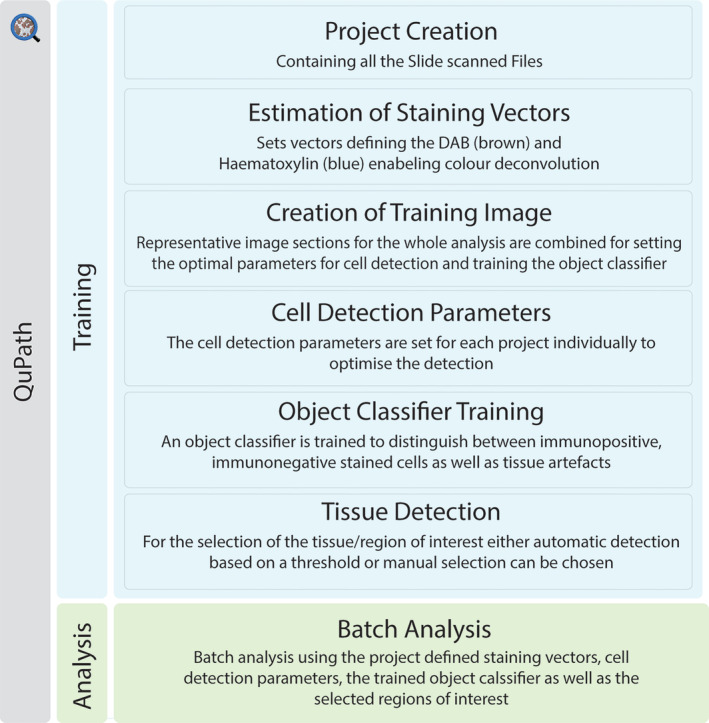
QuPath training. To perform batch analysis with QuPath, the system must first be trained. This involves creating a training project within QuPath that includes all scanned slide files. Staining vectors are then estimated to define the DAB and hematoxylin components, enabling color deconvolution. A representative training image containing various regions reflective of the entire project is selected for training cell detection and object classification. The QuPath Cell Detection plugin is run, and the parameters are adjusted accordingly. Detected cells are manually classified as “positive,” “negative,” or “no” cell. In a new project, regions of interest are selected, either manually by drawing or automatically using a pixel thresholding tool. Once the training process is complete, batch analysis is performed by applying the pre‐determined parameters and values from the training phase to the batch script. This allows consistent analysis across all slides in the project.

In the initial phase of this study, a semi‐automatic pipeline for quantifying low‐cellularity H‐DAB stained tissue slides was developed. Presented as a two‐step tutorial, the H‐DAB semi‐automatic method was designed to extract immunopositivity rates based on cellular classification in different tissues and compute statistical correlation analysis. The study design consisted of four key components: (1) Development of the QuPath pipeline: (a) Samples were stained and digitized, followed by the creation of QuPath projects (Figure 1). (b) Within QuPath, parameters such as deconvolution stain and cell detection were optimized before training a specific object classifier to distinguish between immunopositive and negative cells, as well as tissue artifacts (Figure 2). (c) Batch analysis was performed in regions of interest to detect and classify cells, generating immunopositivity rates. (d) Following QuPath processing, a Python template workflow was developed to enable automatic annotation of QuPath results, integrating data from QuPath, computed immunopositivity statistics, and producing visual outputs such as scatter plots and heatmaps for further analysis. (2) Development of a user guide on GitBook: To ensure the process was accessible and user‐friendly, a detailed step‐by‐step guide was hosted on GitBook [14], outlining how to perform the quantification and calculate the positivity rate for each antibody on the slides. The guide also included troubleshooting tips and best practices for batch analysis. (3) Feedback sessions and survey: The GitBook tutorial was distributed to six testers from four universities with diverse educational backgrounds. Feedback was collected through Zoom calls, where participants shared verbal comments on unclear sections, and through a written survey at the end of the process to gather overall impressions of the pipeline's clarity and usability. (4) Validation: Two evaluations were conducted to validate the pipeline. (a) First, accuracy was validated by comparing semi‐automatic quantification results with manual counting for collagen type II staining and N‐Cadherin staining. (b) Secondly, inter‐rater correlation was assessed by having three raters independently analyze the same set of slides.

### 2.2 Sample Preparation and Digitalization

Frozen or formalin‐fixed paraffin‐embedded sections are suitable for immunohistochemical staining. We conducted DAB (3,3‐Diaminobenzidine) staining according to the immunohistochemical analysis protocol outlined by Binch et al. [2]. Human Intervertebral disc tissue (IVD, Sheffield Research Ethics Committee, IRAS: 10266) and human cartilage (South Yorkshire and North Derbyshire Musculoskeletal BioBank (SYNDMB) REC: 20/SC/0144, 12 182) were collected from Sheffield hospitals). The tissue was fixed in 10% (w/v) formalin (Leica, Milton Keynes, UK), embedded in paraffin wax, sectioned into 4 μm slices using a microtome, and mounted on positively charged slides. The sections were then de‐waxed, rehydrated, and endogenous peroxidases were blocked before antigen retrieval. The cartilage samples were stained for the target antigen interleukin (IL)‐1β (ab9722, 0.5 mg/mL, heat 1:100, Abcam, Cambridge, UK) whereas disc samples were stained for collagen type II (ab3092, enzyme 1:200, Abcam) and N‐Cadherin (ab76011, 0.097 mg/mL, heat 1:100, Abcam). Heat antigen retrieval was performed using 0.05 M Tris (pH 9.5), preheated to 60°C, and incubated for 5 min in a rice steamer. Enzyme antigen retrieval was performed with 0.1% (w/v) α‐chymotrypsin (Sigma Aldrich, Poole, UK) in tris‐buffer saline (TBS, 20 mmol/L Tris, 150 mmol/L NaCl, pH 7.5) containing 0.1% (w/v) CaCl₂ for 30 min at 37°C. Following antigen retrieval washing in TBS, non‐specific antibody binding was blocked for 1 h at room temperature. For IL‐1β and N‐Cadherin, 1% (w/v) bovine serum albumin (BSA) with 25% (v/v) rabbit serum (Sigma) in TBS was used. For collagen type II, 1% (w/v) BSA with 25% (v/v) goat serum in TBS was utilized. Primary antibodies were applied to the slides overnight at 4°C. IL‐1β was used diluted in TBS with 1% (w/v) BSA. IgG controls were used at equal protein concentrations to test for non‐specific binding of the isotype. After overnight incubation, the sections were washed three times in TBS. Secondary antibodies goat anti‐rabbit (ab6720, 1:400, Abcam) or rabbit anti‐mouse IgG (1:400, ab6727, Abcam) were then applied for 30 min at room temperature. Following three washes in TBS, Elite ABC reagent (Vector Laboratories, Peterborough, UK) was added to the slides for 30 min at room temperature. After another three TBS washes, 0.65 mg/mL 3,3′‐diaminobenzidine tetrahydrochloride (Sigma‐Aldrich) containing 0.08% (v/v) H₂O₂ in TBS was added for 20 min. The sections were then washed in running tap water for 5 min. Nuclei were counterstained with hematoxylin for 20 s and blued under running tap water for 3 min. The slides were then dehydrated in graded ethanol, cleared in xylene, and mounted using Pertex (Leica). Slides were scanned at 20× magnification using a slide scanner (PANNORAMIC 250 Flash II DX, 3DHistech, Budapest, Hungary). Slide images scanned in MRXS (Mirax Scan) format (3D HISTECH), NDPI (NanoZoomer Digital Pathology Image) format (Hamamatsu, Shizuoka, Japan), CZI (Carl Zeiss Image) format (Zeiss, Oberkochen, Germany), and BIF (Bio‐Format Image File) format (Ventana, Arizona US) are compatible with this analysis method. Each sample slide was automatically assigned a name during the scanning process, referred to as ID_Slidescanning. This ID_Slidescanning remained unchanged throughout the analysis process to ensure unbiased analysis and was later linked to the sample ID in a subsequent step.

### 2.3 Image Processing in QuPath

For detailed instructions and guide, please visit the QuPath Gitbook guide developed during this project [12, 14, 15, 16, 17, 18, 19, 20, 21]. Key stages and considerations are summarized here (Figure 2).

#### 2.3.1 Project Creation and Estimating of Staining Vectors

A project was created for each stain analyzed to allow for quantification of the whole project automatically, rather than for each scanned sample itself (Figure 2) [14]. The nonspecific binding controls of the isotype were included in the analysis, serving as an accuracy measurement for the analysis. Staining analysis for each antibody was conducted in batches, accommodating between 1 and 100 samples per batch. The quantity of slides that can be processed in each batch relies on the computer's central processing unit (CPU) and random‐access memory (RAM), rather than being determined by the capabilities of QuPath. A separate project is required in QuPath for each antibody and its corresponding regions, containing the relevant images. An algorithm, typically in the form of a simple Plug‐in, is executed within QuPath to deconvolute the color information captured by red‐green‐blue (RGB) cameras. This process estimates the staining vectors, digitally separating the stains and distinguishing between the blue hematoxylin and the brown DAB stain [15]. If multiple regions are analyzed within the same project, the staining vectors should be set for each region separately and saved accordingly.

#### 2.3.2 Cell Detection and Object Classification

**FIGURE 3:**
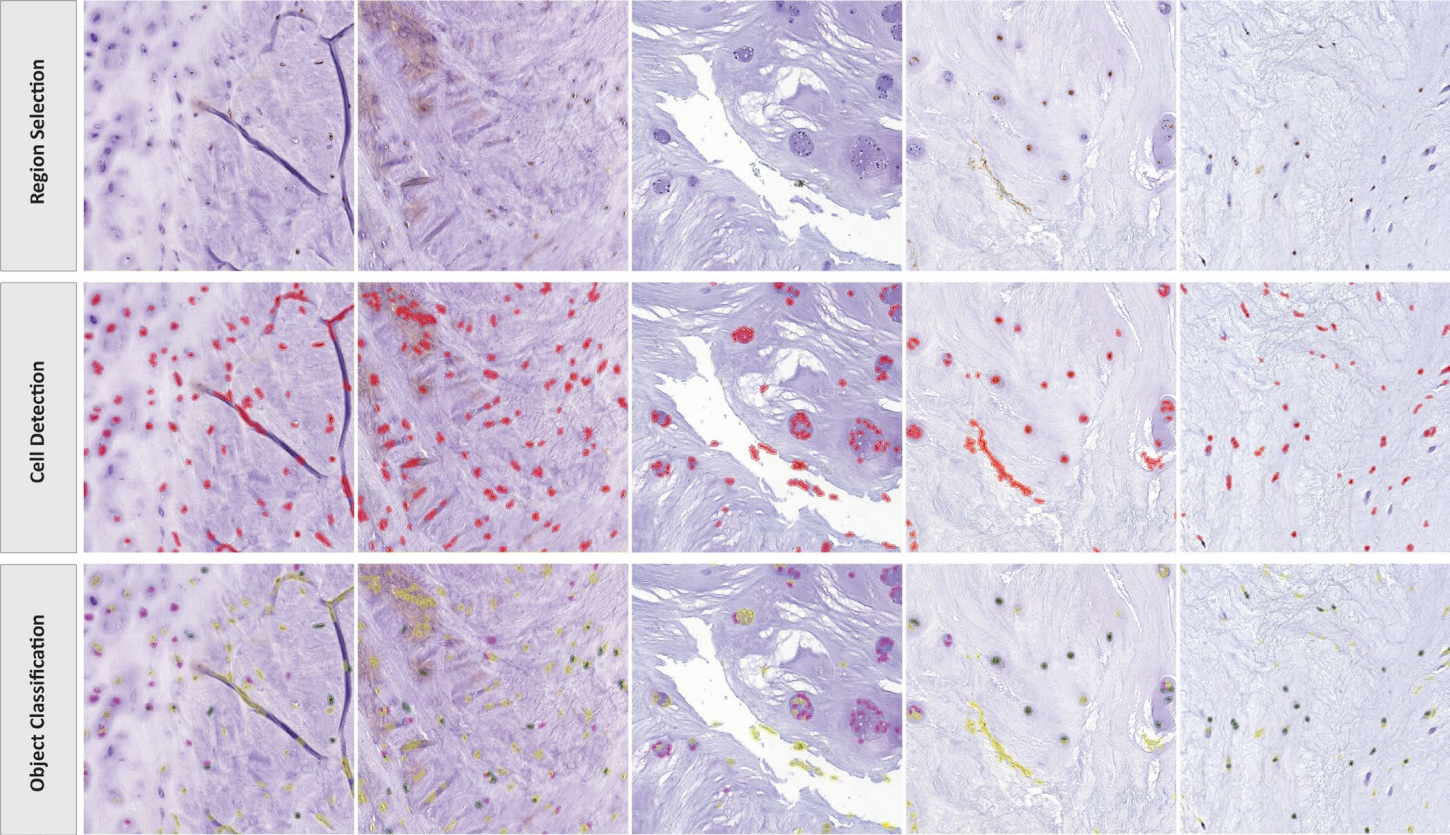
Object classifier. Three stages are completed during the object classifier: Region selection, cell detection, and the object classification. Once a region of interest is selected, the Cell Detection Plugin from QuPath can be run. Shown in red are the detections classified as cells. As the cellularity of the tissue is low and it is quite fibrous, some tissue artifacts are recognized and classified as cells. Once the object classifier, specifically trained for this project, is run, the detected cells are classified as “PositiveCell” (green), “NegativeCell” (pink) and “NoCell” (yellow) enabling the calculation of a positivity rate as the ratio between positively stained cells and the total of negatively and positively stained cells.

QuPath offers an automatic cell detection tool with adjustable parameters to fine‐tune cell detection for each batch [22]. However, in the case of IVD tissue and cartilage, which exhibit a high extracellular matrix to cell ratio, the automatic distinction between positive and negative cells may not be applicable due to the detection of tissue artifacts. To address this issue, an alternative method was developed. As a first step, regions within the tissue are selected to create a training image (Figure 2) [16]. The higher the number of regions included in the training image creation, the more accurate the training. If different components of a tissue are analyzed within one sample, such as the nucleus pulposus and annulus fibrosus within the IVD, training images are created for each component. This is due to anatomical differences, which may result in differences in the optimal parameters for cell detection. As a next step, the cell detection plugin was run on the training image (Figure 3). Parameters such as detection images, requested pixel size, background radius, median filter radius, sigma, minimum area of a nuclei, maximum area of a nuclei, threshold, and maximal background intensity should be adjusted and optimized for each cell detection. Complete details of the influence of these parameters and proposed starting settings for different tissue types are provided in the Gitbook [17], (Table 1). As previously mentioned, some tissue artifacts will be recognized within the software as cells. Thus, training an object classifier was required, which enables classifications of artifacts as “NoCell” and excludes them from the analysis. The Object classifier was manually trained to differentiate between immuno‐positive cells (“PositiveCell”), immuno‐negative cells (“NegativeCell”), and regions devoid of cells (“NoCell”) [18]. Once the precision of the Object classifier was high enough to result in correct measurement of immuno‐positive and ‐negative cells, this was tested on sections and directly corrected, building the accuracy of the Object classifier (Figure 2).

**TABLE 1:** Adjustable parameters for the cell detection.

Parameters	Description	
Detection images	Hematoxylin optical density	Detects nuclei based on the hematoxylin optical density, should only be chosen if DAB staining is not nuclear.
	Optical density sum	Detects nuclei based on the optical density sum, enables detection of nuclei that are masked with DAB, resulting not only in blue but also brown nuclei.
Requested pixel size	Under the Image tab, the pixel size of the slides can be checked, the pixel size should be chosen at the highest value still resulting in accurate results. The bigger the chosen value in pixel size the faster the analysis.	
Background radius	The background radius in QuPath represents the area surrounding each pixel that the software evaluates to estimate the local background intensity. This parameter is essential for accurately differentiating between the cells and tissue and the background. It correlates with the Threshold and should be set greater than the largest nuclei or set to 0 to turn it off. If it is turned off, the threshold needs to be increased	
Median filter radius	The median filter radius is a parameter that serves as noise reduction and smoothening tool. If the nuclei are segmented in the detection, it should be increased.	
Sigma	Sigma refers to the standard deviation parameter used in various filters (commonly Gaussian filter) playing a critical role in determining the smoothening extend of an image.	
Area	The minimum and maximum area of a nuclei is depended on the cell type.	
Threshold	Segments the image by separating objects of interest from the background in a binary way. It can help to remove the detection of false nuclei within the tissue. If a high number of cells is not detected, lowering the threshold is suggested.	
Max background intensity	Refers to the highest intensity value considered to be background. Can remove tissue fold and artifacts, as the background is darker than usual. The lower the value the more folds will be ignored. The Default value doesn't show an effect.	

#### 2.3.3 Tissue Detection/Selection

Even though the training of the object classifier and the tissue detection could be run within the same project, we recommend creating a second project, as it is then easier to make corrections at the end if needed. Under annotations, classifications for the tissue detection were created; these may include, for example, nucleus pulposus, annulus fibrosus, and cartilaginous endplate within IVD tissues, or cartilage and bone within osteochondral tissues [19]. If your sections contain multiple components, the tissue detection needs to be performed manually. To do so, the appropriate classification was selected, and regions of interest were drawn round to be selected for inclusion in counting. If the whole sample consists of only one tissue type, automatic tissue detection can be used (Figure 2). Therefore, a thresholder was created, ignoring all pixels above a certain threshold. If the staining within the project varies, it is important to ensure all the slides are checked and adjusted either manually or by altering the thresholder. The thresholder was saved as “TissueDetection” If there were visible tissue folds, they were removed from the detection so that they did not result in false data [19].

#### 2.3.4 Batch Analysis

To perform the batch image analysis, a script was written which is freely available and downloadable [20, 21]. The parameters for the estimation of the staining vectors and the parameters for the cell detection were manually replaced for each project, creating a project‐specific script for each region and antibody of interest. Scripts were then run in a text file and automatically saved in a new results folder within the QuPath project folder. The text file contains information for each of the parent regions, e.g., cartilage, the detected objects and their type, e.g., cells, and the classification of the cells (Positive/Negative).

### 2.4 Process Scanning Data—Python Package

QuPath [5] is the gold standard software used in biological image analysis, even though it falls short in automating data processing. To export the results and represent statistical measurements for each sample, in this step‐by‐step tutorial we developed a novel tool designed to automate the post‐processing of data from various whole‐slide image formats, known as ProcessScanningData Python class [12]. This was run as a second step following the generation of files from the scanned images processed through the aforementioned method in QuPath.

The Python class, built up with specific libraries, such as NumPy [23] and MatLab [24], is customizable for MRXS, NDPI, CZI, and BIF files. Merging these image‐derived data with inventory files organizes it into output files in either XLSX or CSV, depending on the user's preference. This results in automatic and precise calculation of positivity rates and produces visualizations for every individual marker [13]. To unveil their potential biological correlation and significance, scatterplots and heatmaps were produced with Python libraries [25] based on standard statistical coefficients between positivity rates for different markers (e.g., Pearson, Spearman, and Kendall correlation coefficients) [26, 27, 28]. In the tutorial, rather than using the Python class directly, the focus is on a customizable workflow template tailored to user preferences; for example, it is employed for MRXS files in this study. Notably, the dedicated Gitbook section [12] provides a guide for users through the entire process, from software installation to the final generation of results.

#### 2.4.1 Usage of Process Scanning Data in IHC Data Analysis

The project has used the ready‐to‐use workflow and personalized it for analyzing immunopositivity within histological images, as defined by the previous method in QuPath. A step‐by‐step description from inputs to outputs is provided in the section ‘Processing’ in the tutorial [12].

Each input file is processed to extract detailed cellular and tissue‐level metrics, such as object IDs, classifications (e.g., Positive/Negative Cell), region coordinates, and detailed nucleus and cell measurements (e.g., area, perimeter, circularity, and optical density of hematoxylin and DAB stains), generated from the QuPath analysis. The processed data is then further analyzed to calculate each marker's positivity rates. The results are compiled into structured output files (e.g., CSV or XLSX), which include essential details such as Sample ID, Antibody, Image, Positive/Negative Class, and Positivity Rate. The final output consists of processed data files or structured files (e.g., CSV or XLSX) containing detailed information on cell metrics, positivity rates, and respective metadata, and correlation analysis files or visual representations (heatmaps and scatterplots) that illustrate the relationships between different biomarkers, providing a comprehensive and organized dataset that researchers can use for further analysis or publication. An additional merging step can be added to the pipeline, which aims to create a final spreadsheet, linking the donor/sample information with the positivity rates of the antibodies within specific regions, to end up with a single comprehensive file for further observation and analysis.

## 3 Method Evaluation

### 3.1 Manual Versus Semiautomatic Counting

A single evaluator assessed 28 IVD samples, 14 stained for Collagen type II and 14 stained for N‐Cadherin, both manually and following the semi‐automatic guidelines provided by QuPath. The similarity between the results obtained from the two methods was analyzed using a paired *t*‐test (*p* ≤ 0.05), and correlation was assessed using a Pearson Correlation in GraphPad Prism (Version 10.3.1509, Windows, GraphPad Software, Boston, Massachusetts USA).

### 3.2 Interrater Variability

Three evaluators from the University of Sheffield conducted an analysis to determine the positivity rate for 17 IL‐1β immunohistochemically stained slides, following the same pipeline. The results were analyzed using the intraclass correlation coefficient (ICC) for single measures, based on a two‐way mixed model with absolute agreement, in SPSS Statistics (IBM Corp. Released 2023. IBM SPSS Statistics for Windows, Version 29.0.2.0 Armonk, NY: IBM Corp). While two of the evaluators had prior experience with the pipeline (raters 2&3), the third was performing the analysis and using QuPath for the first time (rater 1). The evaluators varied in academic rank, comprising a PhD student, a postdoctoral researcher, and a professor.

### 3.3 Survey

A survey was conducted to assess the clarity of the GitBook documentation and the level of satisfaction with the results obtained using the semi‐automatic QuPath method. Participants were contacted via email, either directly or through their principal investigator, and were provided with an information letter outlining the study and inviting them to participate in the evaluation of the semi‐automatic quantification guide. After using the GitBook, participants assessed various aspects of the guide's usability using a Likert scale ranging from 1 (strong dissatisfaction) to 10 (strong satisfaction) (Supplementary Table S1). A total of six participants from the University of Bern (Switzerland), Icahn School of Medicine at Mount Sinai (USA), the University of Arizona College of Medicine (USA), and the University of Sheffield (UK) participated in the survey. The group included two medical students, one PhD student, two postdoctoral researchers, and one professor.

## 4 Results

### 4.1 Manual Versus Semi‐Automatic Counting

**FIGURE 4:**
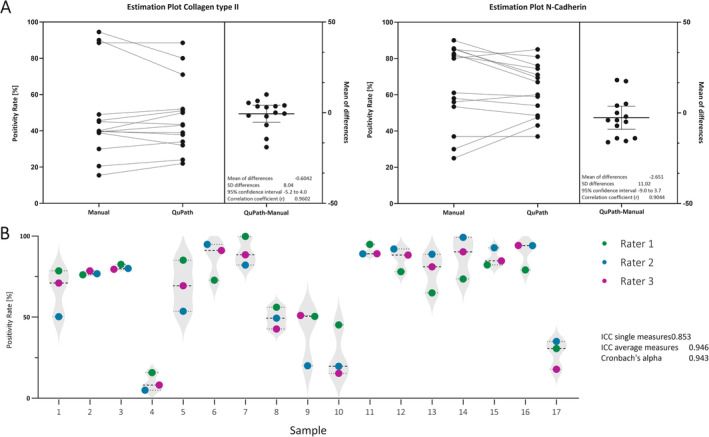
Method evaluation. (A) Graph showing a paired *t*‐test of the immunopositivity rates for immunohistochemically stained slides for Collagen type II (*n* = 14) and N‐Cadherin (*n* = 14) analyzed by a single rater manually and with QuPath. For the positivity rate of collagen type II staining, the mean of differences between the manual and QuPath quantification was −0.604, with a standard deviation of 8.04. The correlation coefficient between the two methods was 0.960. For the positivity rate of N‐Cadherin staining, the mean of differences was −2.65, with a standard deviation of 11.02. The correlation coefficient between the manual and semi‐automatic QuPath‐based quantification was 0.904. (B) Graph displaying the positivity rates obtained in 17 slides by 3 different Raters indicated by different colors. The positivity rates differed between the raters, with a maximal standard deviation of 14.5 and a minimal standard deviation of 1. The Intraclass correlation for single measures was 0.853, and for average measures, it was 0.946. Cronbach's alpha was 0.943.

A two‐tailed *t*‐test revealed no significant differences between the semi‐automatic QuPath method and manual counting for either collagen type II staining (*p* = 0.783) or N‐cadherin staining (*p* = 0.386) (Figure 4A). For collagen type II staining, the mean difference between the methods was −0.6042, with a standard deviation (SD) of 8.039 and a 95% confidence interval of −5.245–4.038. Similarly, for N‐cadherin staining, the mean difference was −2.651, with an SD of 11.02 and a 95% confidence interval of −9.016–3.71 (Figure 4A). The data pairing was highly effective, as indicated by strong correlation coefficients for both collagen type II (*r* = 0.9602, *p* < 0.0001) and N‐cadherin (*r* = 0.9044, *p* < 0.0001), suggesting that the two methods are closely aligned. For both antibodies, the effect size was small (*R* ^2^ = 0.006 for collagen type II and *R* ^2^ = 0.0586 for N‐cadherin), indicating minimal variability between the semi‐automatic QuPath method and manual counting. The average cell count for the manual counting was 200, while the average cell count for the QuPath analysis on these samples was 600.

### 4.2 ICC Between 3 Raters

**FIGURE 5:**
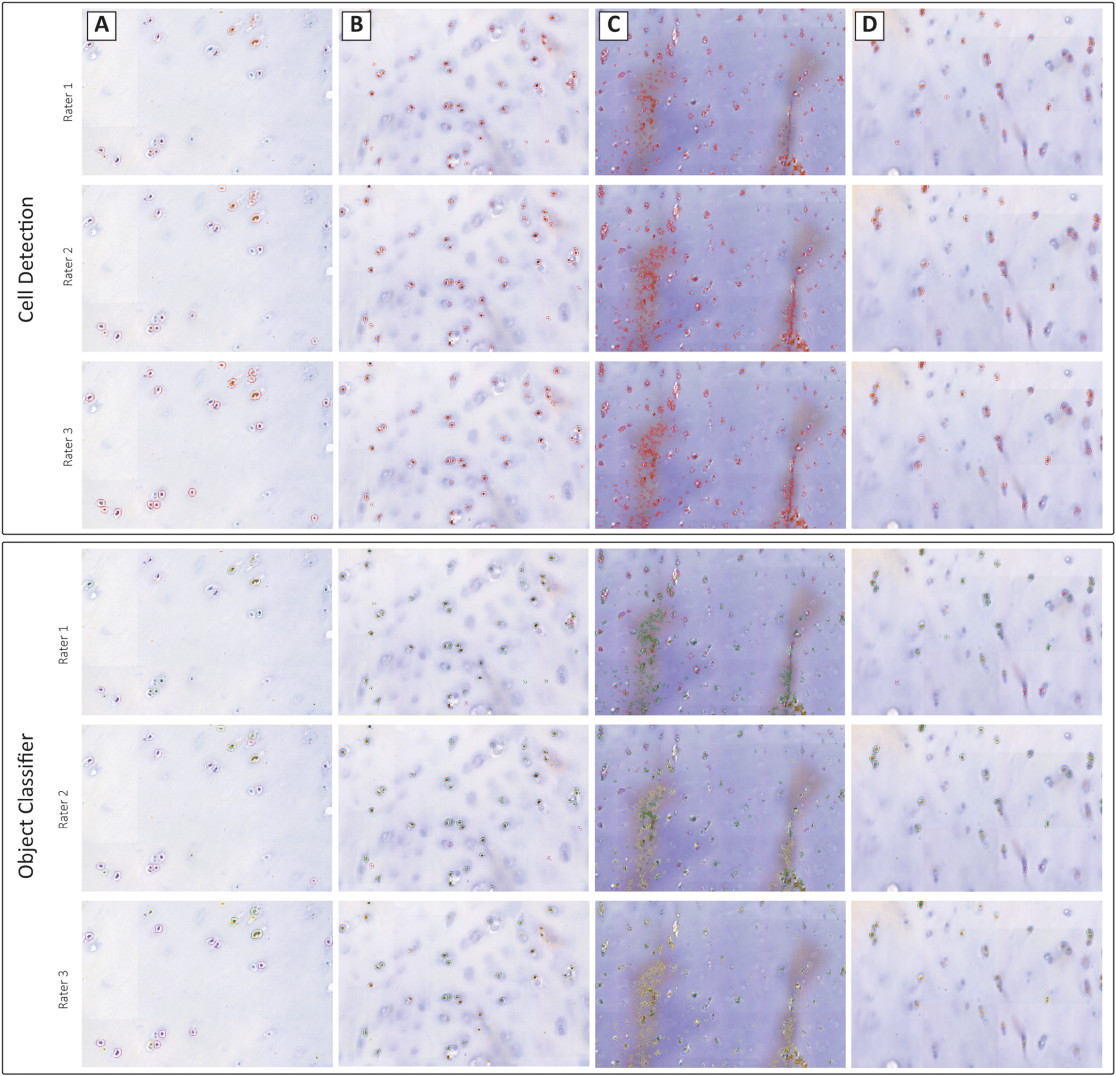
Examples of microscopic images to explain reasons for interrater differences in cell detection and object classification. Differences between the different raters in parameters set for the cell detection as well as for the object classification. The top box shows the cell detection; detected objects are marked in red. The bottom box shows the object classification, displaying immunopositively classified cells in green, immune‐negatively classified cells in pink, and objects to ignore in yellow. While images in row A and B show high similarities in the detection and classification, images C and D differ highly. Rater 1 (row C) has not trained the classifier to exclude and ignore DAB debris within the stain, while rater 2 partly ignores it, and rater 3 fully. To acquire exact measurements, rater 1 would therefore need to exclude mal‐stained regions from the analysis. Row D shows an image that is blurred. Rater 1 classifies blurred cells as positive and negative, while rater 3 classifies them as no cells and excludes them from the analysis.

The ICC for single measures was 0.853, with 95% confidence intervals ranging from 0.706 to 0.939. For average measures, the ICC was 0.946, with a 95% confidence interval between 0.878 and 0.979. The inter‐item correlation matrix showed that rater 1 and rater 2 showed a correlation coefficient of 0.781, rater1 and rater3 0.892; rater 2 and rater 3 0.923, demonstrating the closest correlation between the two raters with prior experience of IHC analysis (Figure 4B). Differences in the positivity rates were traced back to differences in the QuPath training, the manual selection of the tissue, but also the quality of the staining (Figure 5). The slides showing greater inter‐rater variability showed a high background/noise staining with DAB, complicating the identification of cells and their classification (Figure 5).

### 4.3 Survey Evaluation

**FIGURE 6:**
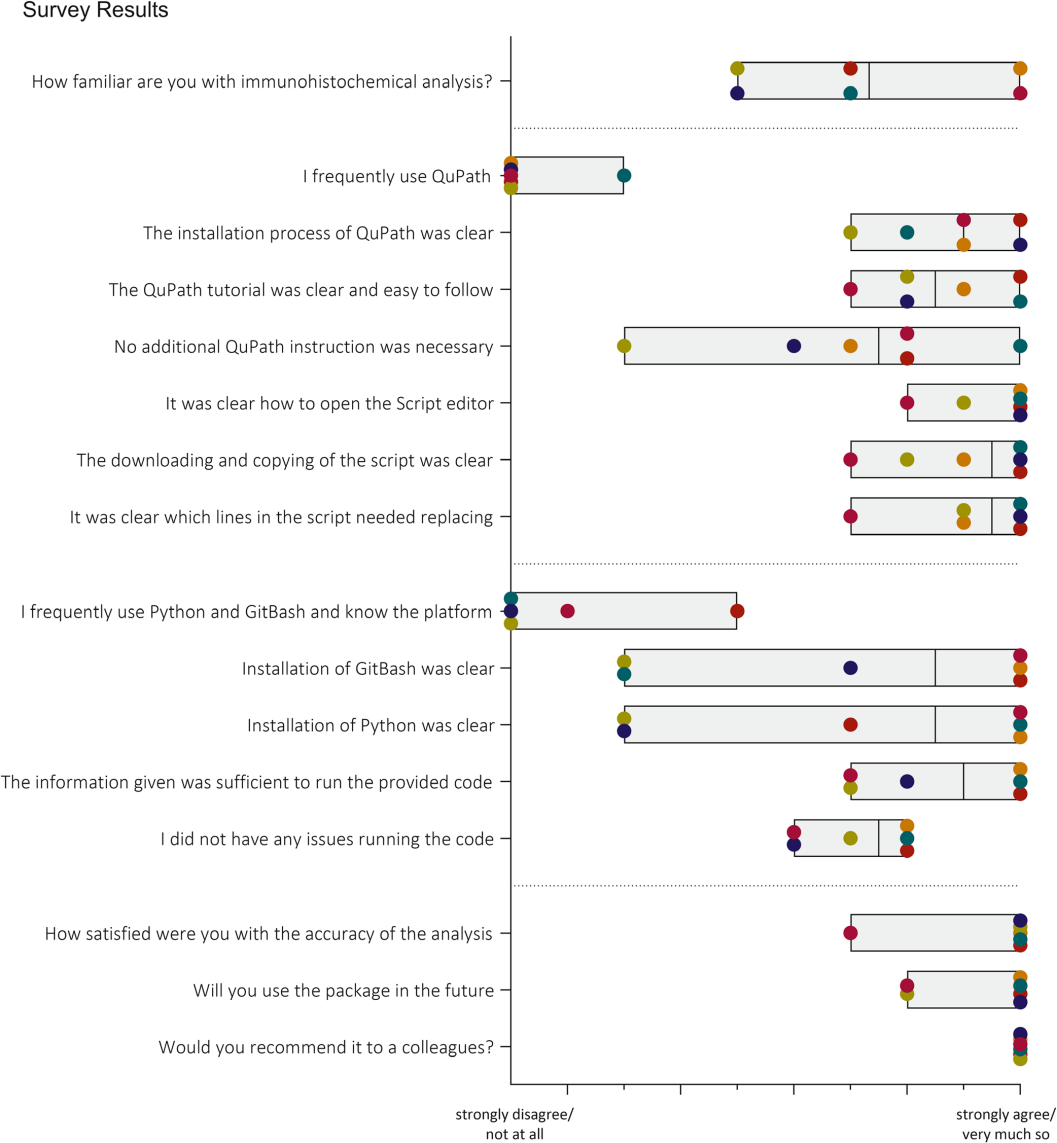
Survey evaluation results: Participants assessed various aspects of the guide's usability using a Likert scale ranging from 1 (strong dissatisfaction) to 10 (strong satisfaction). The different floating bars range from the minimal rated value to the maximum rated value, showing the mean as a line. The different colors represent the different raters, demonstrating general satisfaction in the Qu Path processing guide and ease of use of processing code, and agreement that all participants would recommend its use to a colleague.

Survey evaluation showed that five out of six participants had no prior experience with QuPath, while one had used it occasionally (Figure 6). Their familiarity with immunohistochemical analysis ranged from slightly familiar (*n* = 2) to extremely familiar (*n* = 2). The clarity of the installation process received an average score of 8.83/10, and the QuPath tutorial was rated at 8.67/10 (Figure 6). Some participants sought additional help from the official QuPath webpage, which was linked in the GitBook. The tasks of downloading, opening the script, accessing the script editor, and modifying the script lines were rated highly, with scores of 9/10, 9.5/10, and 9.17/10, respectively. Only two participants had prior experience with Python and GitBash, but the installation process was straightforward for Windows users. However, Mac users gave the process a lower rating of 3/10 (Figure 6).

The information provided in the GitBook was rated as sufficient for running the Python script (8.67/10), and participants were able to resolve issues independently while executing the code (Figure 6). One participant analyzed 10–20 slides, four analyzed 20–30 slides, and one participant analyzed over 50 slides using the provided script. The tissues analyzed included IVD tissue from humans and rats, as well as osteochondral tissue and cartilage. While one participant was somewhat satisfied with the detection and classification of cells in QuPath, five participants reported being extremely satisfied (Figure 6). Regarding future use, two participants indicated they would probably use the package, and four participants stated they would definitely use it. All participants reported that they would recommend it to a friend or colleague (Figure 6).

## 5 Discussion

IHC is a well‐established and widely accepted method in both clinical and experimental medical sciences for assessing the localization and semi‐quantification of proteins. However, the commonly used manual approach of counting 200 cells is limited by human fatigue, subjective interpretation of color intensity, and regional bias, all of which reduce the accuracy and reproducibility of this method [29, 30]. In response, several research fields [31], particularly in cancer research [3, 4, 6], have developed semi‐automatic or fully automated methods, emphasizing the need for standardized, accurate, scalable, and reproducible evaluation techniques. However, these methods are not suitable for tissues like the IVD or cartilage due to morphological differences and the low cellularity of these tissues.

This study aimed to develop a guide for semi‐automatic quantitative analysis of IHC‐stained slides from low‐cellularity tissues using QuPath. A fully automated pipeline was not implemented due to the region‐dependent nature of cell detection parameters [32], which requires the system to be trained to differentiate between immunopositive and immunonegative cells, as well as tissue artifacts. The semi‐automatic approach also allows for flexibility, enabling the use of various immunohistochemical staining protocols. The focus of this study was specifically on cellular immunopositivity, and the quantification of extracellular matrix (ECM) staining was beyond the scope of our analysis. While techniques such as area positivity measurements could be employed to assess ECM staining, quantifying staining intensity for ABC/DAB staining is not appropriate due to amplification of the staining and, therefore, potential inaccuracies in intensity or area‐based quantification [2].

The method's evaluation involved comparing manual and semi‐automatic results, along with calculating inter‐rater correlation coefficients from the same sample set, following the established guidelines. The high correlation between manual and semi‐automatic counting confirmed the accuracy and highlighted the robustness of the semi‐automatic QuPath method. Slight differences in positivity rates were attributed to the higher cell count and reduced regional bias in QuPath's quantification. The ICC analysis demonstrated strong reliability for both single raters and averaged measures, underscoring the robustness of the method. The single‐measure ICC indicates good agreement among individual raters, while the higher ICC for average measures reflects excellent reliability when scores from multiple raters are averaged. This highlights the value of incorporating multiple raters to enhance the precision of measurements [33]. The inter‐item correlation matrix further supports these findings, showing high consistency between raters, particularly between raters 2 and 3 who had experience with IHC analysis previously, while rater 1 exhibited slightly lower agreement with the others, suggesting a potential learning curve. These differences in inter‐rater reliability were attributed to variations in QuPath training, manual tissue selection, and staining quality. Slides with greater inter‐rater variability were characterized by high background staining with DAB, which made cell identification and classification more challenging. These findings highlight the importance of standardized training and stringent quality control during staining and image preparation to minimize variability. Overall, the results confirm that while individual raters achieve good reliability, averaging ratings from multiple raters substantially enhances the consistency and robustness of the method, particularly when addressing challenges posed by staining artifacts or subjective variability in tissue selection. The survey results indicated that the instructions provided in the GitBook were clear and sufficient for users to successfully follow and perform the analysis, yielding highly satisfactory outcomes in cell detection and classification. Importantly, all participants stated they would recommend it to a colleague, and most participants expressed an intention to use the method for future analyses.

Considering QuPath as the benchmark for biological image analysis, it lacks automation for tasks includingcalculating positivity rates and correlation coefficients, as well as generating customized visualizations [5]. As a result, users have been forced to export the results and manually process the MRXS results, occasionally using other tools like Excel or R, which for non‐experts can be time‐consuming and error‐prone. Additionally, manual data processing limits scalability when dealing with large datasets.

The ProcessScanningData class, designed with a versatile hierarchical structure, offers a robust solution. It can serve as the foundation for a customized workflow, tailored to the type of scanned files and the user's expertise level in bioinformatics. Accompanied by a template and extended documentation, the instantiation of a customizable workflow is simplified for both experts and non‐experts, ultimately producing the output files necessary for the assessment of immunopositivity and the generation of scatter plots and heatmaps [11, 12] A standardized method like ProcessScanningData is crucial because it ensures reproducibility and portability across studies, avoiding the inconsistencies and arbitrary interpretations that can arise with manual curation. By eliminating subjective calculations, it provides a reliable framework for exploring relationships between different biomarkers, making it an invaluable resource for researchers working with immunohistological data.

The proposed pipeline for semi‐automatic counting demonstrates consistent and reliable performance, as evidenced by the high ICC values observed in this study. A comparison between manual and semi‐automatic counting demonstrated that both methods yielded similar results, with the semi‐automatic approach offering a more streamlined process by utilizing pre‐defined parameters. However, the dataset for this comparison was relatively small, and ICC analysis was not performed for manual counting by multiple raters, which limits the ability to fully evaluate variability in the manual method. While the semi‐automatic pipeline reduces the need for repetitive, labor‐intensive tasks, human input during the training phase and manual tissue selection can still introduce variability. Variability observed in slides with high background staining or poorly defined tissue regions highlighted the importance of standardized training and quality control to ensure reliable results across both methods.

## 6 Limitations

The methods used in this analysis have several limitations. First, the requirement of a slide scanner limits accessibility, as not all laboratories may have the necessary equipment. Additionally, the poor staining quality of the slides can negatively impact the accuracy of both manual and semi‐automatic counting, as unclear or inconsistent staining makes cell identification more difficult. Furthermore, there is an element of subjectivity in the classification process, especially when training the semi‐automatic system, which can introduce variability in results despite the use of standardized parameters. These factors must be considered when interpreting the data. Finally, during the evaluation process, multiple users reported issues while using a Mac. CaseViewer, used for the visualization of 3D HISTECH scanned slides, is not available on Macs; however, 3DHistech Panoramic viewer can be downloaded for free in the App Store for iPads.

## 7 Conclusion

In conclusion, this study presents a semi‐automatic pipeline for quantitative analysis of immunohistochemically stained slides, particularly for low‐cellularity tissues such as IVDs and cartilage. The semi‐automatic method developed using QuPath has been proposed to address key limitations of manual cell counting, such as human fatigue, subjective interpretation, and regional bias, with the aim of improving accuracy and reproducibility across groups following uptake. High inter‐rater agreement and strong correlation with manual counting underscore the method's robustness and reliability. Furthermore, the ProcessScanningData class enhances the workflow by providing a scalable, customizable solution for processing large datasets, reducing manual intervention, and facilitating reproducible results. Despite minor reliance on manual training for cell detection, this approach represents a significant step forward in standardizing IHC analysis, making it a valuable tool for researchers dealing with immunohistochemical data where the outcome measure of interest is cellular immunopositivity. The positive feedback from users, who reported high satisfaction and intent to continue using the method, with all survey participants reporting that they would recommend its use to colleagues, highlights its practical applicability and potential for widespread adoption in future studies.

## Conflicts of Interest

The authors declare no conflicts of interest.

## Supporting information


**Table S1.** Survey.

## References

[1] L. D. True , “Quality Control in Molecular Immunohistochemistry,” Histochemistry and Cell Biology 130 (2008): 473–480, 10.1007/s00418-008-0481-0. 18648842 PMC2522330

[2] A. Binch , J. Snuggs , and C. L. Le Maitre , “Immunohistochemical Analysis of Protein Expression in Formalin Fixed Paraffin Embedded Human Intervertebral Disc Tissues,” JOR Spine 3, no. 3 (2020), 10.1002/JSP2.1098. PMC7524243 33015573

[3] M. B. Loughrey , P. Bankhead , H. G. Coleman , et al., “Validation of the Systematic Scoring of Immunohistochemically Stained Tumour Tissue Microarrays Using QuPath Digital Image Analysis,” Histopathology 73, no. 2 (2018): 327–338, 10.1111/HIS.13516. 29575153

[4] S. Di Cataldo , E. Ficarra , A. Acquaviva , and E. Macii , “Automated Segmentation of Tissue Images for Computerized IHC Analysis,” Computer Methods and Programs in Biomedicine 100, no. 1 (2010): 1–15, 10.1016/J.CMPB.2010.02.002. 20359767

[5] P. Bankhead , M. B. Loughrey , J. A. Fernández , et al., “QuPath: Open Source Software for Digital Pathology Image Analysis,” Scientific Reports 7, no. 1 (2017): 16878, 10.1038/S41598-017-17204-5. 29203879 PMC5715110

[6] K. R. Choudhury , K. J. Yagle , P. E. Swanson , K. A. Krohn , and J. G. Rajendran , “A Robust Automated Measure of Average Antibody Staining in Immunohistochemistry Images,” Journal of Histochemistry and Cytochemistry 58, no. 2 (2010): 95–107, 10.1369/JHC.2009.953554. 19687472 PMC2803710

[7] C. T. Thorpe and H. R. C. Screen , “Tendon Structure and Composition,” Advances in Experimental Medicine and Biology 920 (2016): 3–10, 10.1007/978-3-319-33943-6_1. 27535244

[8] A. A. Biewener , “Tendons and Ligaments: Structure, Mechanical Behavior and Biological Function,” in Collagen: Structure and Mechanics, ed. P. Fratzl (Springer, 2008), 10.1007/978-0-387-73906-9_10.

[9] A. J. Sophia Fox , A. Bedi , and S. A. Rodeo , “The Basic Science of Articular Cartilage: Structure, Composition, and Function,” Sports Health 1, no. 6 (2009): 461–468, 10.1177/1941738109350438/ASSET/IMAGES/LARGE/10.1177_1941738109350438-FIG5.JPEG. 23015907 PMC3445147

[10] B. R. Whatley and X. Wen , “Intervertebral Disc (IVD): Structure, Degeneration, Repair and Regeneration,” Materials Science and Engineering: C 32, no. 2 (2012): 61–77, 10.1016/J.MSEC.2011.10.011.

[11] M. P. Ferri , “Processing Scanning Data,” https://mapoferri/processscanningdata.

[12] A. Nüesch and M. P. Ferri , “QuPath Guide for Semi‐Automatic Quantification of Immunohistochemically Stained Slides‐ Processing Package Tutorial Gitbook,” https://disc4all‐qupath.gitbook.io/qupath‐project/result‐analysis‐docs/processing‐package‐tutorial.

[13] C. R. Taylor and R. M. Levenson , “Quantification of Immunohistochemistry—Issues Concerning Methods, Utility and Semiquantitative Assessment II,” Histopathology 49, no. 4 (2006): 411–424, 10.1111/J.1365-2559.2006.02513.X. 16978205

[14] A. Nüesch and M. P. Ferri , “QuPath Guide for Semi‐Automatic Quantification of Immunohistochemically Stained Slides,” https://disc4all‐qupath.gitbook.io/qupath‐project.

[15] A. Nüesch and M. P. Ferri , “QuPath Guide for Semi‐Automatic Quantification of Immunohistochemically Stained Slides‐Estimating Staining Vectors,” https://app.gitbook.com/o/kAkxf5RLoV6dm2APAW3P/s/SleK316zl0BYwa7DfK2J/qupath‐h‐dab‐docs/qupath‐h‐dab‐tutorial/estimating‐stain‐vectors.

[16] A. Nüesch and M. P. Ferri , “QuPath Guide for Semi‐Automatic Quantification of Immunohistochemically Stained Slides‐Training Image Creation,” https://app.gitbook.com/o/kAkxf5RLoV6dm2APAW3P/s/SleK316zl0BYwa7DfK2J/qupath‐h‐dab‐docs/qupath‐h‐dab‐tutorial/training‐image‐creation.

[17] A. Nüesch and M. P. Ferri , “QuPath Guide for Semi‐Automatic Quantification of Immunohistochemically Stained Slides‐Cell Detection,” https://disc4all‐qupath.gitbook.io/qupath‐project/qupath‐h‐dab‐docs/qupath‐h‐dab‐tutorial/cell‐detection.

[18] A. Nüesch and M. P. Ferri , “QuPath Guide for Semi‐Automatic Quantification of Immunohistochemically Stained Slides‐Object Classifier,” https://app.gitbook.com/o/kAkxf5RLoV6dm2APAW3P/s/SleK316zl0BYwa7DfK2J/qupath‐h‐dab‐docs/qupath‐h‐dab‐tutorial/object‐classifier.

[19] A. Nüesch and M. P. Ferri , “QuPath Guide for Semi‐Automatic Quantification of Immunohistochemically Stained Slides‐Tissue Detection,” https://app.gitbook.com/o/kAkxf5RLoV6dm2APAW3P/s/SleK316zl0BYwa7DfK2J/qupath‐h‐dab‐docs/qupath‐h‐dab‐tutorial/tissue‐detection.

[20] A. Nüesch , “H‐DAB Batch Analysis Script,” https://github.com/anuesch/Disc4All_QuPath_H‐DAB_Script.

[21] A. Nüesch and M. P. Ferri , “QuPath Guide for Semi‐Automatic Quantification of Immunohistochemically Stained Slides‐Batch Analysis Script,” https://app.gitbook.com/o/kAkxf5RLoV6dm2APAW3P/s/SleK316zl0BYwa7DfK2J/qupath‐h‐dab‐docs/qupath‐script.

[22] P. Bankhead , M. B. Loughrey , J. A. Fernández , et al., “QuPath: Open Source Software for Digital Pathology Image Analysis,” Scientific Reports 7, no. 1 (2017): 1–7 Accessed December 2, 2023, https://pubmed.ncbi.nlm.nih.gov/29203879/. 29203879 10.1038/s41598-017-17204-5 PMC5715110

[23] C. R. Harris , K. J. Millman , S. J. van der Walt , et al., “Array Programming With NumPy,” Nature 585, no. 7825 (2020): 357–362, 10.1038/s41586-020-2649-2. 32939066 PMC7759461

[24] W. McKinney , “Data Structures for Statistical Computing in Python,” Proceedings of the 9th Python in Science Conference 445 (2010): 56–61, 10.25080/MAJORA-92BF1922-00A.

[25] J. D. Hunter , “Matplotlib: A 2D Graphics Environment,” Computing in Science & Engineering 9, no. 3 (2007): 90–95, 10.1109/MCSE.2007.55.

[26] K. V. I. I. Pearson , “Note on Regression and Inheritance in the Case of Two Parents,” Proceedings of the Royal Society of London 58, no. 347–352 (1895): 240–242, 10.1098/RSPL.1895.0041.

[27] C. Spearman , “The Proof and Measurement of Association Between Two Things,” American Journal of Psychology 15, no. 1 (1904): 72, 10.2307/1412159. 3322052

[28] M. G. Kendall , “A New Measure of Rank Correlation,” Biometrika 30, no. 1/2 (1938): 81, 10.2307/2332226.

[29] A. De Gramont , S. Watson , L. M. Ellis , et al., “Pragmatic Issues in Biomarker Evaluation for Targeted Therapies in Cancer,” Nature Reviews. Clinical Oncology 12, no. 4 (2015): 197–212, 10.1038/NRCLINONC.2014.202. 25421275

[30] F. Aeffner , K. Wilson , N. T. Martin , et al., “The Gold Standard Paradox in Digital Image Analysis: Manual Versus Automated Scoring as Ground Truth,” Archives of Pathology & Laboratory Medicine 141, no. 9 (2017): 1267–1275, 10.5858/ARPA.2016-0386-RA. 28557614

[31] A. L. Hein , M. Mukherjee , G. A. Talmon , et al., “QuPath Digital Immunohistochemical Analysis of Placental Tissue,” Journal of Pathology Informatics 12, no. 1 (2021): 40, 10.4103/JPI.JPI_11_21. 34881095 PMC8609285

[32] P. Bankhead , “Developing Image Analysis Methods for Digital Pathology,” Journal of Pathology 257, no. 4 (2022): 391–402, 10.1002/PATH.5921. 35481680 PMC9324951

[33] T. K. Koo and M. Y. Li , “A Guideline of Selecting and Reporting Intraclass Correlation Coefficients for Reliability Research,” Journal of Chiropractic Medicine 15, no. 2 (2016): 155–163, 10.1016/J.JCM.2016.02.012. 27330520 PMC4913118

