# Supplementary material for: Application and Validation of Semiautomatic Quantification of Immunohistochemically Stained Sections for Low Cellular Tissue Such as Intervertebral Disc Using QuPath
Source: JOR Spine. 2025 Mar 6;8(1):e70054. doi: 10.1002/jsp2.70054 (PMC11885164; doi:10.1002/jsp2.70054)
Supplement: Supplementary file 1 — Table S1. Survey. [file JSP2-8-e70054-s001.docx]

Supplementary Table 1 | Survey

| **P1** |
| --- |
| **What institution are you from?**  University of Bern |
| University of Sheffield |
| Icahn School of Medicine at Mount Sinai |
| University of Sheffield |
| University of Arizona College of Medicine |
| The University of Sheffield |
|  |
| **P2** |
| **What is your highest degree?** |
| Postgraduate degree |
| Doctoral degree |
| Undergraduate degree |
| Doctoral degree |
| Undergraduate degree |
| Doctoral degree |
|  |
| **P3** |
| **What is your role in the institution?** |
| PhD Student |
| Professor/PI |
| Other please state: |
| PostDoc |
| Other please state: |
| PostDoc |
|  |
| **P4** |
| **How familiar are you with immunohistochemical analysis?** |
| Moderately familiar |
| Extremely familiar |
| Slightly familiar |
| Moderately familiar |
| Slightly familiar |
| Extremely familiar |
|  |
| **P5** |
| **Have you ever used QuPath previously?** |
| Never |
| Never |
| Never |
| Sometimes |
| Never |
| Never |
|  |
| **GB1_4** |
| **Please rate your experience with the QuPath H-DAB tutorial provided. - I frequently use QuPath and know the platform** |
| 9 |
| 5 |
| 9 |
| 4 |
|  |
| **GB1_1** |
| **Please rate your experience with the QuPath H-DAB tutorial provided. - The installation process of QuPath was clear** |
| 10 |
| 9 |
| 7 |
| 8 |
| 10 |
| 9 |
|  |
| **GB1_2** |
| **Please rate your experience with the QuPath H-DAB tutorial provided. - The QuPath tutorial was clear and easy to follow.** |
| 10 |
| 9 |
| 8 |
| 10 |
| 8 |
| 7 |
|  |
| **GB1_3** |
| **Please rate your experience with the QuPath H-DAB tutorial provided. - No additional QuPath instruction was necessary** |
| 8 |
| 7 |
| 3 |
| 10 |
| 6 |
| 8 |
|  |
| **GB1.1** |
| **What was unclear about the installation?** |
| Some steps were difficult to translate from windows to mac. |
|  |
| **GB1.3** |
| **What else would have been needed?** |
| Mac-specific instructions would have been great! |
| Maybe instructions for mac vs. PC |
|  |
| **GB2_1** |
| **Please rate your experience with the QuPath H-DAB script - It was clear how to open the Script editor** |
| 10 |
| 10 |
| 9 |
| 10 |
| 10 |
| 8 |
|  |
| **GB2_2** |
| **Please rate your experience with the QuPath H-DAB script - The downloading and copying of the script was clear** |
| 10 |
| 9 |
| 8 |
| 10 |
| 10 |
| 7 |
|  |
| **GB2_3** |
| **Please rate your experience with the QuPath H-DAB script - It was clear which lines in the script needed replacing** |
| 10 |
| 9 |
| 9 |
| 10 |
| 10 |
| 7 |
|  |
| **GB2.2** |
| **What was unclear?** |
| How to change some of the scripts and how to use them |
|  |
| **GB2.3** |
| **What else would you have needed?** |
| Clearer instructions on how to use them |
|  |
| **GB3_5** |
| **Please rate your experience with GitBash and Python - I frequently use python and GitBash and know the platform** |
| 5 |
| 1 |
| 1 |
| 1 |
| 2 |
|  |
| **GB3_1** |
| **Please rate your experience with GitBash and Python - Installation of GitBash was clear** |
| 10 |
| 10 |
| 3 |
| 3 |
| 7 |
| 10 |
|  |
| **GB3_2** |
| **Please rate your experience with GitBash and Python - Installation of Python was clear** |
| 7 |
| 10 |
| 3 |
| 10 |
| 3 |
| 10 |
|  |
| **GB3_3** |
| **Please rate your experience with GitBash and Python - The information given was sufficiant to run the provided code** |
| 10 |
| 10 |
| 7 |
| 10 |
| 8 |
| 7 |
|  |
| **GB3_4** |
| **Please rate your experience with GitBash and Python - I did not have any issues running the code** |
| 8 |
| 8 |
| 7 |
| 8 |
| 6 |
| 6 |
|  |
| **GB3.1.2** |
| **What was unclear?** |
| 1)During Cell Classifier step it took me a bit to realise how to choose NoCell/PossitiveCell/NegativeCell option while I was classifying  2) I missed the info of installing certain Python packages |
| Like previous steps, mac-specific instructions would have been great. |
|  |
| **GB3.4** |
| **What was the error message?** |
| Most issues were regarding the installation process, not running the script within QuPath |
|  |
| **E1** |
| **What kind of tissue did you use for the analysis?** |
| Human IVD tissue |
| IVD & Cartilage |
| Rat spine |
| Osteochondral tissue |
| IVD |
| Intervertebral disc |
|  |
| **Q35** |
| **How many slides have you analysed** |
| 20-30 |
| 50+ |
| 20-30 |
|  |
| 20-30 |
| 20-30 |
|  |
| **E3** |
| **How satisfied were you with the detection and classification of the script?** |
| Extremely satisfied |
| Extremely satisfied |
| Extremely satisfied |
| Extremely satisfied |
| Extremely satisfied |
| Somewhat satisfied |
|  |
| **Batch Y N** |
| **Did you perform the analysis on the provided training batch?** |
| Yes |
| Yes |
| No |
| Yes |
| Yes |
| No |
|  |
| **E3.1** |
| **Does anything need to be improved regarding the detection and classification of the script?** |
| No :) |
| no |
| No |
|  |
| **E4** |
| **Will you use the QuPath script and analysis package in the future?** |
| Definitely yes |
| Definitely yes |
| Probably yes |
| Definitely yes |
| Definitely yes |
| Probably yes |
|  |
| **E5** |
| **Will you recommend the QuPath script and analysis package to a friend or colleague?** |
| Definitely yes |
| Definitely yes |
| Definitely yes |
| Definitely yes |
| Definitely yes |
| Definitely yes |
|  |
| **Q36** |
| **Do you have any furhter suggestions or comments?** |
| Further is spelt incorrectly on this question. |
| None, thank you! |
| No |
| No, thank you! |
| Make instructions slightly clearer for each step and how to put the correct codes into Git Bash |
